# Tumeur glomique de la cuisse: nouveau cas et revue de la littérature

**DOI:** 10.11604/pamj.2017.28.73.12827

**Published:** 2017-09-26

**Authors:** Omar Margad, Nabile Bousselmame

**Affiliations:** 1 Service de Traumatologie-Orthopédie de l’Hôpital Militaire Avicenne, Marrakech, Maroc; 2 Cabinet Médical, Appartement 12, Immeuble 23, Avenue Bin al Auidane, Agdal, Rabat, Maroc

**Keywords:** Tumeur glomique, cuisse, douleur, Glomus tumor, thigh, pain

## Abstract

Les tumeurs glomiques sont rares et bénignes qui se développent au-dépends du corps glomique neuromyoartériel. Si la localisation digitale est bien connue en chirurgie de la main, les localisations exradigitales souffrent d'une méconnaissance aboutissant à des erreurs diagnostiques et thérapeutiques. Nous rapportons un nouveau cas de tumeur glomique de la cuisse, et à travers une revue de la littérature, nous essayerons d'attirer l'attention vers ces localisations atypiques.

## Abstract (en)

Glomus tumors are rare and benign. They originate from the neuromyoarterial glomus body. Digital glomus tumors are a well-known condition in hand surgery. On the contrary, there is a lack of knowledge with regard to extradigital glomus tumor resulting in diagnostic and therapeutic errors. We here report a new case of glomus tumor of the thigh. Our literature review aims to focus attention on these uncommon locations.

## Introduction

Les tumeurs glomiques sont des tumeurs bénignes et rares qui se développent aux-dépends du glomus neuromyoartériel des anastomoses artério-veineuses. Elles touchent surtout les extrémités digitales, mais les localisations extra digitales ne sont pas rares mais surtout méconnues ce qui est responsable du retard de leur diagnostic et de leur prise en charge. Le but de notre travail est de rapporter un nouveau cas de tumeur glomique de la cuisse, et d'attirer l'attention vers cette localisation inhabituelle souvent méconnue de douleurs inexpliquées de la cuisse.

## Patient et observation

**Figure 1:**
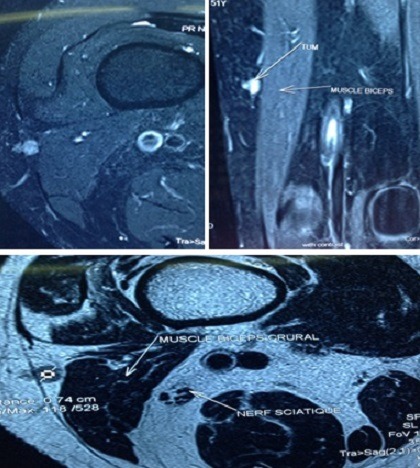
Résultats IRM objectivant une masse ovale sous aponévrotique hypo intense en T1, hyper intense en T2, siégeant à l’extérieur du long biceps fémoral

**Figure 2:**
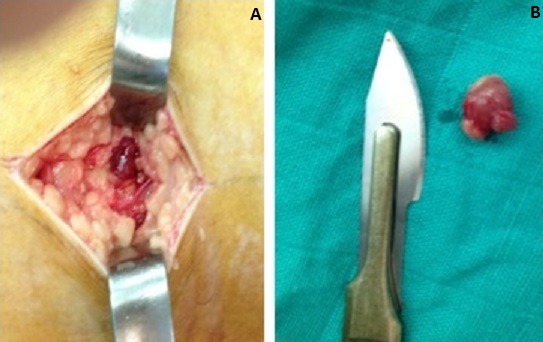
A) image per opératoire de la tumeur glomique; B) image après exérèse

Il s'agit d'un patient âgé de 40 ans, sans antécédents pathologiques particuliers que nous avons reçus après avoir fait le tour de plusieurs médecins de spécialités différentes et après échec des traitements symptomatiques et de rééducation fonctionnelle. Ce patient présentait des douleurs du tiers moyen de la cuisse droite évoluant depuis 3 ans. Au début, ces douleurs étaient paroxystiques, déclenchées par le toucher ou après une activité sportive de moyenne intensité, ensuite ces douleurs devenaient permanentes, s'aggravant à la flexion du genou. L'examen clinique de ce patient montrait une boiterie à la marche, une amyotrophie de la cuisse droite chiffrée à 1,5 cm, une douleur provoquée au niveau de la région postéro externe de la cuisse à un endroit ou le patient précisait bien. Aussi, avons-nous constaté une exacerbation de la douleur lors de la flexion. Les examens complémentaires ont comporté une radiographie du fémur qui est revenu normal, une IRM (Figure 1), qui a objectivé une masse ovale hypo intense en T1, rehaussée après injection de Gadolinium, hyper intense en T2, siégeant au niveau du tiers moyen de la cuisse derrière le long biceps fémoral. Une biopsie exérèse (Figure 2) a été réalisée et l'examen anatomopathologique a confirmé le diagnostic de glomique .Les suites opératoires ont été simples, le patient a eu un soulagement immédiat de douleur et l'examen clinique du patient après un recul de 2 ans a trouvé un patient asymptomatique avec récupération de la trophicité de la cuisse.

## Discussion

Les tumeurs glomiques sont rares et bénignes, elles représentent environ 1 à 5% de l'ensemble des tumeurs des tissus mous [1]. Ce sont des tumeurs de petite taille, qui touchent le sujet adulte, l'âge moyen est de 40 ans, elles sont rares avant 20 ans [2]. Signalée pour la première fois par Wood en 1812 [3] qu'il a dénommé (nodule sous cutané douloureux), c'est Masson [4] en 1924 qu'il l'a définit en décrivant le glomus qu'il nomme ainsi à cause de sa similitude avec la glande vasculaire coccygienne de Luschka [5]. Sa symptomatologie associe typiquement une triade caractéristique: 1) douleur provoquée, le patient peut préciser le siège exact de la lésion; 2) douleur spontanée, qui apparait tardivement avec l'évolution, 3) douleur d'hypersensibilité thermique, avec un contraste entre l'intensité des signes subjectifs et la pauvreté des signes objectifs. Si la localisation digitale est bien connue en chirurgie de la main [6], les localisations atypiques [7–10] souffrent de méconnaissance, des études ont montré que seulement 9 à 20% des patients ont eu un diagnostic correct dès le départ [11, 12], ce qui conduit à des erreurs diagnostiques et thérapeutiques. Il n'y a pas d'imageries spécifiques permettant la confirmation diagnostique, cependant l'échographie, malgré sa faible spécificité aide à localiser la lésion [13]. L'IRM reste le gold standard dans le diagnostic des tumeurs glomiques, elle précise le siège exact de la lésion et sa relation avec les structures avoisinantes [14–16]. Son traitement est toujours chirurgical, il consiste en une exérèse chirurgicale qui entraine une disparition rapide des douleurs. Les récidives sont rares mais possibles, elles sont toujours précoces et le fait d'exérèse incomplète, d'où la recommandation de certains auteurs [17–19] d'exciser plus que les limites apparentes de la tumeur.

## Conclusion

Les tumeurs glomiques sont rares mais non exceptionnelles. Elles peuvent siéger partout ou existe les glomi. Devant toute douleur avec ou sans masse palpable, sans étiologie évidente, le diagnostic de tumeur glomique doit être évoqué.

## Conflits d’intérêts

Les auteurs ne déclarent aucun conflit d'intérêt.
